# Algal and Fungal Antioxidants Alleviate Oxidative Stress‐Induced Reproductive Defects

**DOI:** 10.1002/fsn3.70301

**Published:** 2025-05-16

**Authors:** Nazli Pinar Arslan, Seyda Albayrak, Aysenur Budak‐Savas, Ahmet Hacimuftuoglu, Tugba Orak, Aysenur Ozdemir, Omer Karadagoglu, Sevval Yildirim, Handan Cinar‐Yilmaz, Mesut Taskin

**Affiliations:** ^1^ Vocational School of Health Services Bingol University Bingol Turkey; ^2^ Department of Molecular Biology and Genetics, Faculty of Science Ataturk University Erzurum Turkey; ^3^ Department of Medical Pharmacology, Faculty of Medicine Ataturk University Erzurum Turkey; ^4^ Vaccine Development Application and Research Center Ataturk University Erzurum Turkey

**Keywords:** antioxidants oxidative stress, infertility, testis ovarian

## Abstract

The intrinsic and extrinsic factors (natural aging, diseases, infections, chemicals, radiation, heavy metals etc.) create oxidative stress, thereby causing reproductive defects in males and females. Similarly, oxidative stress causes the abnormalities in sperm and oocytes, and thus reduces the success of in vitro fertilization. Fungi and/or algae‐derived metabolites (polysaccharides, carotenoids, ergothioneine, phycobiliproteins, phlorotannins, and cordycepin) alleviate the damages in ovaries and testis and correct the impaired reproductive functions (spermatogenesis, ovulation, fertilization and implantation) in the in vivo models by increasing antioxidant enzyme activities (superoxide dismutase, catalase, glutathione peroxidase etc.), making mitochondrial parameters better (membrane potential, distribution, dynamics and activity of mitochondria), decreasing oxidative stress (the reductions in intracellular ROS and malondialdehyde levels), inflammation (the reductions in COX‐2, IL‐6, IL‐1β, and TNF‐α levels) and apoptosis (the reductions in *Caspase‐3* and *Bax* levels) and balancing blood hormone levels (follicle stimulating hormone, luteinizing hormone, testosterone). Similarly, in the in vitro models, they increase antioxidant enzymes activities, decrease oxidative stress and apoptosis, and make mitochondrial functions better, thereby restoring the defects in sperm and oocyte parameters and increasing blastocyst formation. Overall, this review study reveals that the antioxidants from fungi and algae contribute to the improvement of the reproductive outcome of humans and animals and assisted reproductive technology by preventing or alleviating oxidative stress. However, more in vitro, in vivo, or clinical studies are needed to prove the safety profile and efficacy of fungi‐ and algae‐based antioxidants. This is the first review study focusing on the protective effects of fungi and algae‐based antioxidants against oxidative stress‐induced reproductive defects.

## Graphical abstract

In the in vivo models, fungi and/or algae‐derived metabolites (polysaccharides, carotenoids, ergothioneine, phycobiliproteins, phlorotannins, and cordycepin) correct the impaired reproductive functions in males and/or females by increasing antioxidant enzyme activities, decreasing oxidative stress, inflammation, and apoptosis, alleviating ovarian and testis damages, and balancing blood hormone levels. In the in vitro models, they increase antioxidant enzyme activities, decrease oxidative stress and apoptosis, and improve mitochondrial functions, thereby restoring the defects in sperm and oocyte parameters and increasing blastocyst formation.

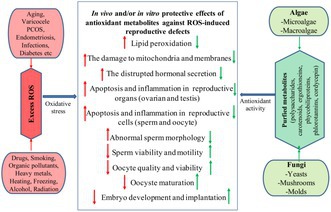

**Abbreviations**
CAT: catalase
COX‐2: cyclooxygenase 2
GPx: glutathione peroxidase
GSH: glutathione
H_2_O_2_
: hydrogen peroxide
IL‐1β: interleukin‐1 beta
IL‐6: interleukin 6
IVM: in vitro maturation media
*MDA*
: *malondialdehyde*
NO^•^
: nitric oxide radical
O_2_^•–^
: superoxide radical
OH^•^
: hydroxyl radical
ROS: reactive oxygen species
SIRT1: sirtuin 1
SOD: superoxide dismutase
TNF‐α: tumor necrosis factor‐alpha

## 1 Introduction

In living systems including humans, reactive oxygen species (ROS) at adequate levels are indispensable for the proper functions of various cellular processes, whereas excess accumulation of ROS damages cellular molecules, membranes, and organelles, thereby causing undesired health problems, such as infertility, neurodegenerative diseases, cardiometabolic disorders, inflammatory bowel disease, cancer, diabetes, and natural physiological aging (Arslan et al. 2022, 2023; Arslan, Taskin, and Keles 2024; Arslan, Orak, et al. 2024; Esim et al. 2024). For instance, ROS at adequate levels are vital for the proper functions of reproductive system organs and cells in humans, whereas excess ROS, namely oxidative stress, is one of the primary reasons for human infertility. ROS‐induced oxidative stress causes DNA and membrane damage in sperm, reduces sperm motility and viability, sperm acrosome reaction, and sperm‐oocyte interaction, thereby negatively influencing male infertility (Lopes et al. 2021; Arslan et al. 2022; Takeshima et al. 2021; Mannucci et al. 2022). Similarly, oxidative stress causes ovarian damage in females, thereby reducing oocyte quality and maturation, blastocyst formation, and implantation (Agarwal et al. 2005; Wang et al. 2021; Yan et al. 2022; Arslan, Taskin, and Keles 2024).

In the literature, it has been documented that the oxidative stress created by intrinsic or extrinsic factors can cause impaired functions and damage in the organs (testis, ovaries, uterine etc.) and cells (sperm, oocytes, etc.) of reproductive systems in females and males. Especially natural physiological aging is considered the main factor responsible for the reduced ovarian functions and fertility in females (Alchalabi et al. 2016; Gao et al. 2017; Harlev et al. 2017; Ramgir and Abilash 2019; Abudawood et al. 2021; Santacruz‐Márquez et al. 2021; Arslan et al. 2022; Madhu et al. 2022; Zhang et al. 2023; Arslan, Taskin, and Keles 2024).

To date, the in vivo Works have revealed that natural or synthetic exogenous antioxidants can protect reproductive system organs and cells against intrinsic and extrinsic oxidative stress factors. Similarly, the exogenous antioxidants can also protect sperm, oocytes, or embryos against oxidative stress factors in the in vitro models (Nabenishi et al. 2012; Walczak‐Jedrzejowska et al. 2013; Ahmad et al. 2017; Yu et al. 2019; Shahat et al. 2020; Ghorbani et al. 2021; Vašková et al. 2023; Arslan, Taskin, and Keles 2024). For example, the potential of plant‐derived antioxidant metabolites (phenolics, alkaloids, anthocyanins, polysaccharides etc.) or their extracts to alleviate oxidative stress‐induced reproductive defects has been revealed by numerous research articles (Li et al. 2015; Shi et al. 2018; Bahramrezaie et al. 2019; Soleimanzadeh et al. 2020; Jalili et al. 2021; Dong et al. 2024). For example, Bahramrezaie et al. (2019) found that resveratrol, a plant‐derived phenolic, improved oocyte and blastocyst quality when orally applied to humans. Shi et al. (2018) found that the polysaccharide purified from the plant *Lycium barbarum* alleviates diabetic testicular dysfunction by inhibiting oxidative stress‐induced abnormal autophagy in male mice. A recent work performed by Dong et al. (2024) elucidated that anthocyanins from the plant *L. ruthenicum* murray could activate the keap1/nrf2 signaling pathway and thus attenuated cadmium‐induced oxidative stress and testicular toxicity. Furthermore, the protective potential of plant‐derived metabolites or their extracts against reproductive defects has been discussed in some review articles (Sorelle et al. 2019; Akbaribazm et al. 2021; Yang et al. 2021; Vašková et al. 2023). On the contrary, this potential of the metabolites (polysaccharides, carotenoids, ergothioneine, phycobiliproteins, phlorotannins, and cordycepin) from fungi and/or algae has not been mentioned in any review article yet. Therefore, this review focuses on intensifying the current literature knowledge about the in vitro and/or in vivo protective influences of these antioxidative metabolites on reproduction system organs and cells of humans and animals. This review study was prepared by considering mostly the publications of the last 5 years (2020–2024).

## 2 Antioxidants Against Oxidative Stress‐Induced Diseases

Reactive oxygen species (ROS) are a set of unstable molecules generated by all kinds of cells. The most well‐known ROS are hydrogen peroxide (H_2_O_2_), hydroxyl radical (OH^•^), singlet oxygen (^1^O_2_) and superoxide (O_2_^•–^) (Su et al. 2019). Endogenous sources of ROS are mitochondria, endoplasmic reticulum, peroxisomes, nuclei, cytosol, plasma membranes, and extracellular spaces. The main source of mitochondrial ROS is the electron transport chain located on the inner mitochondrial membrane. Therefore, ROS are accepted as byproducts of aerobic energy metabolism in cells (Bedard and Krause 2007). The majority of intracellular ROS is generated by mitochondria; however, the endoplasmic reticulum (ER), which generates about 25% of total ROS, is considered another important contributor of ROS (Tu and Weissman 2004). Moreover, there are multiple exogenous factors leading to ROS production in the cells, such as air pollutants, tobacco smoke, ionizing and nonionizing radiations, drugs, xenobiotics, heavy metals, and pesticides (Bhattacharyya et al. 2014).

In living organisms, ROS at adequate levels (low or moderate level) functions as signal transducers, thereby mediating different cellular processes (cell proliferation, migration, differantion, apoptosis etc.). Besides, adequate levels of ROS can prevent pathogens. Therefore, adequate levels of ROS are termed as “good stress.” While over‐accumulation of ROS in cells creates oxidative stress (bad stress) and the detrimental influences in cells by oxidizing cellular molecules (nucleic acids, proteins, and lipids) (Liguori et al. 2018; Lee and Song 2021; Nakamura and Takada 2021; Jomova et al. 2023; Esim et al. 2024). For example, high ROS levels cause disruption of energy production in mitochondria, inhibiting respiratory system enzymes. Similarly, high ROS levels cause the disruption of mitochondrial dynamics by affecting mitochondrial fission and fusion proteins (Damas et al. 2014; Lakshmanan et al. 2018). The exposure of lipids to ROS results in lipid peroxidation. This event harms phospholipids directly and can also behave as a cell signal inducing programmed cell death. Furthermore, oxidized phospholipids can mediate proinflammatory changes (Que et al. 2018). For instance, membranes of cells or organelles are especially more susceptible to the detrimental influences of ROS since they have a higher content of polyunsaturated fatty acids (Arslan et al. 2023; Esim et al. 2024).

ROS‐induced oxidative stress, namely bad‐stress, is involved in pathological conditions, such as infertility, cancer, inflammatory bowel disease, diabetes, rheumatoid arthritis, cardiovascular vascular diseases (hypertension, atherosclerosis, heart failure, and atrial fibrillation), retinal diseases (macular degeneration, glaucoma, diabetic retinopathy, and retinal vein occlusion), neurodegenerative diseases (Alzheimer's disease, Parkinson's disease, Huntington's Disease and Wilson Disease), renal dysfunction, chronic kidney disease, obesity, natural aging, and skin aging (Popolo et al. 2013; Damas et al. 2014; Bhatti et al. 2017; Mao et al. 2017; Masuda et al. 2017; Lakshmanan et al. 2018; Nebbioso et al. 2022; Arslan et al. 2022; Arslan, Taskin, and Keles 2024). Accordingly, maintaining ROS at adequate levels, namely low or moderate levels, is indispensable for performing cellular functions and preventing oxidative stress. In humans, this equilibrium was maintained by endogenous and exogenous molecules with antioxidant activity.

The general endogenous antioxidant system consists of two groups of antioxidants: antioxidant enzymes and non‐enzymatic antioxidants. The first group contains superoxide dismutase (SOD), catalase (CAT) and glutathione peroxidase (GPx), and thioredoxin (Trx), whereas the second group includes coenzyme Q10, melatonin, bilirubin, glutathione, metal‐binding proteins, etc. (Mirończuk‐Chodakowska et al. 2018; Arslan et al. 2023).

Exogenous antioxidants are mainly taken with diet or as supplements from plants; however, they can be obtained from other natural sources, including algae, lichens, bacteria, and fungi. The antioxidants derived from these natural sources exhibit protective roles towards oxidative stress‐caused diseases (Mirończuk‐Chodakowska et al. 2018; Ak Sonat et al. 2018; Liang et al. 2019; Jędrejko et al. 2021; Khavari et al. 2021; Ampofo and Abbey 2022; Arslan et al. 2023; Chen et al. 2023; Vignaud et al. 2023; Naik and Gupte 2023; Rahim et al. 2024).

## 3 Antioxidants Against Oxidative Stress‐Induced Reproductive Defects

Infertility is defined as the insufficiency to accomplish spontaneous pregnancy in spite of regular unprotected sexual intercourse for 12 months or longer. This disease affects about 15% of couples (Mannucci et al. 2022). Oxidative stress is one of the major factors of male and female infertility (Agarwal et al. 2005; Arslan et al. 2022).

Physiological levels of ROS, usually described as low or moderate ROS levels, are essential for sperm maturation and motility as well as its chemotaxis, capacitation, hyperactivation, acrosome reaction, and oocyte interaction properties (Mannucci et al. 2022). On the contrary, excess ROS damages the reproductive organs and cells in males and females. For instance, ROS‐induced oxidative stress causes the histomorphological abnormalities in the tunica albuginea, germ cells, seminiferous tubules, and interstitial tissue of testis (Dutta et al. 2021; Arafa et al. 2024). Furthermore, excess ROS changes the fluidity and integrity of sperm membrane by causing lipid peroxidation in membranes, thereby altering sperm morphology and decreasing motility and viability. Eventually, functional and morphological abnormalities prevent the sperms from impregnating the ovum, thereby causing infertility (Chandra et al. 2012; Chianese and Pierantoni 2021; Momeni and Eskandari 2020; Hussain et al. 2023). Furthermore, ROS‐provoked oxidative stress significantly increases malondialdehyde (MDA) levels in seminal plasma by causing lipid peroxidation (Dorostghoal et al. 2017).

In females, the ovaries have two main functions. The first is to produce hormones that activate the female reproductive system, and the second is to control oocyte development, selection, and release through the process known as folliculogenesis (Barnett et al. 2006). Adequate levels of ROS are essential for steroidogenesis, folliculogenesis, ovulation, tubal function, implantation, and embryo development (Kaltsas et al. 2023). Conversely, ROS‐induced oxidative stress leads to histomorphological abnormalities in oocytes, ovaries, and the uterus, and also negatively affects plasma hormonal levels (Sharma et al. 2015; Alchalabi et al. 2016; Arslan, Taskin, and Keles 2024). Moreover, oxidative stress expedites ovarian aging and enhances apoptosis, inflammation, mitochondrial damage, telomere shortening, and macromolecular damage, thereby causing the loss or decrease of ovarian functions (folliculogenesis, ovulation, implantation etc.; Agarwal et al. 2005; Wang et al. 2021; Yan et al. 2022; Arslan, Taskin, and Keles 2024).

**FIGURE 1:**
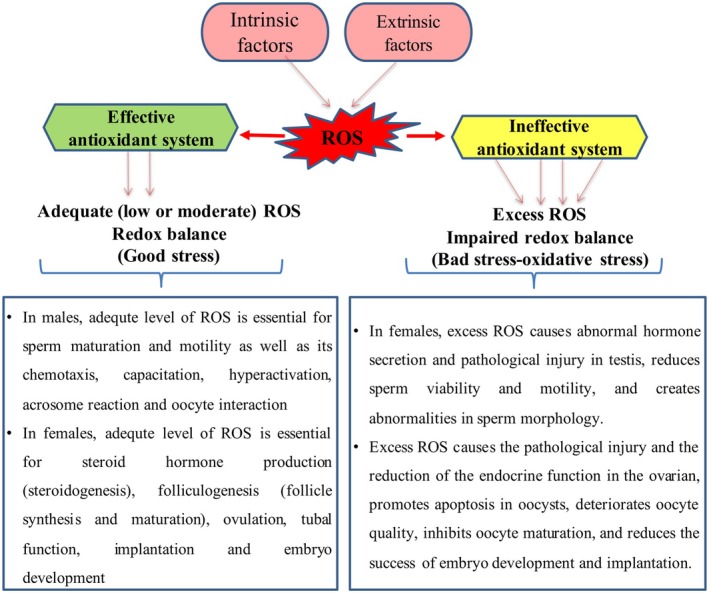
The balance between antioxidants and reproductive parameters.

Natural physiological aging is one of the major contributors to female infertility. Although physiological aging affects all body organs, the aging process in the ovaries occurs faster than in other organs, such as the uterus, pituitary gland, or pancreas (Amanvermez and Tosun 2016; Arslan, Taskin, and Keles 2024). During the aging process, oxidative stress that occurs with ROS accumulation reduces the quality of oocytes in the ovaries, provokes apoptosis of granulosa cells, accelerates degeneration of the corpus luteum, reduces communication between oocytes and granulosa cells, and impedes oocyte maturation (Sasaki et al. 2019; Cajas et al. 2020; Yang et al. 2021). Besides, the number of developing embryos decreases since ovarian aging reduces oocyte quality. Moreover, as the endocrine function of the ovary decreases with aging, the abnormalities in the uterine vascular system occur, which reduces the success of implantation (Gougeon et al. 1994; Szafarowska and Jerzak 2013; Meldrum et al. 2016; Ansere et al. 2021). In addition to natural aging, some diseases (endometriosis, polycystic ovarian syndrome, varicocele, infections etc.) and other exogenous factors (chemo‐therapeutic agents, radiation, cigarette smoking, organic pollutants, heavy metals, alcohol use etc.) can cause ROS accumulation and oxidative stress in males and females, and eventually animal and human infertility (Gao et al. 2017; Harlev et al. 2017; Ramgir and Abilash 2019; Abudawood et al. 2021; Santacruz‐Márquez et al. 2021; Arslan et al. 2022; Madhu et al. 2022; Zhang et al. 2023; Arslan, Taskin, and Keles 2024; Figure 1).

Overall, it can be said that oxidative stress negatively affects spermatogenesis, ovulation, and implantation in animals and humans. Therefore, preventing excessive ROS accumulation is considered an important approach in protecting the reproductive system organs and cells of females and males against oxidative stress. In this context, using the antioxidants from natural sources (plant, fungi, macroalgae, microalgae, bacteria etc.) is considered a solution for the reduction or prevention of oxidative stress‐related reproductive abnormalities.

## 4 Biotechnological Importance of Fungi and Algae

Fungi are eukaryotic organisms that are distributed in the terrestrial and aquatic environments. They are categorized into three major groups: single‐celled yeasts, filamentous micro‐fungi (molds), and macroscopic filamentous fungi (mushrooms). Only a small part of fungi is pathogenic, and their majority is advantageous for humans. For instance, yeasts, molds, and mushrooms are used to produce bioactive substances that find diverse applications in food, cosmetic, nutraceutical, and pharmaceutical industries (Arslan et al. 2023; Arslan, Orak, et al. 2024).

Algae are single or multicellular photosynthetic organisms living in water or in humid environments and producing their own food. Algae are generally divided into microalgae and macroalgae. The size of microalgae varies from 1 mm to several cm, and they can be only visible through magnification equipment, whereas the size of macroalgae can reach up to 60 m in length, and they can be perfectly visible to the naked eye (Biris‐Dorhoi et al. 2020; Pereira 2021). On the basis of their pigmentation, macroalgae can be categorized into three major groups: brown seaweed (*Phaeophyceae*), red seaweed (*Rhodophyceae*), and green seaweed (*Chlorophyceae*) (Deepika et al. 2022).

Macroalgae, also known as seaweed, have high contents of carbohydrate, protein, lipid, vitamin, and fiber. They are also rich in essential amino acids and minerals. Due to the rich nutritional composition, some macroalgae are consumed as food or food additives for humans or animals (Deepika et al. 2022; Guo et al. 2022). Besides, they produce different polysaccharides (agar, alginate, carrageenan etc.), which find significant usages in food, cosmetic, and pharmaceutical industries (Chudasama et al. 2021; Deepika et al. 2022). Moreover, macroalgae can synthesize secondary metabolites exhibiting diverse biological activities, and they are therefore important for pharmaceutical applications (Silva et al. 2020).

Microalgae have an eukaryotic or a prokaryotic cell structure. Prokaryotic microalgae are grouped into *Cyanophyta* and *Prochlorophyta* divisions, whereas the eukaryotic microalgae include the following divisions: *Chlorophyta, Euglenophyta, Rhodophyta, Haptophyta (Prymnesiophyta), Heterokontophyta (Bacillariophyceae, Chrysophyceae, Xantophyceae*, among others), *Cryptophyta*, and *Dinophyta* (Silva et al. 2019). Microalgae exist in unicellular as well as multicellular forms, and have the potency to live in aquatic and terrestrial environments. They are widely employed as bioremediation agents in wastewater‐treatment systems for eliminating heavy metals, dyes, toxic gases, and petroleum‐based contaminants (Esim et al. 2024). Microalgae possessing high lipid content are used as feedstock for biodiesel production (Maity et al. 2014). Some microalgae are consumed as food by humans. Furthermore, microalgae have biotechnological and medicinal importance since they are able to produce natural metabolites possessing promising biological activities (Khavari et al. 2021; Ampofo and Abbey 2022).

## 5 Fungi‐And Algae‐ Derived Antioxidants Against Oxidative Stress‐Induced Reproductive Defects

**FIGURE 2:**
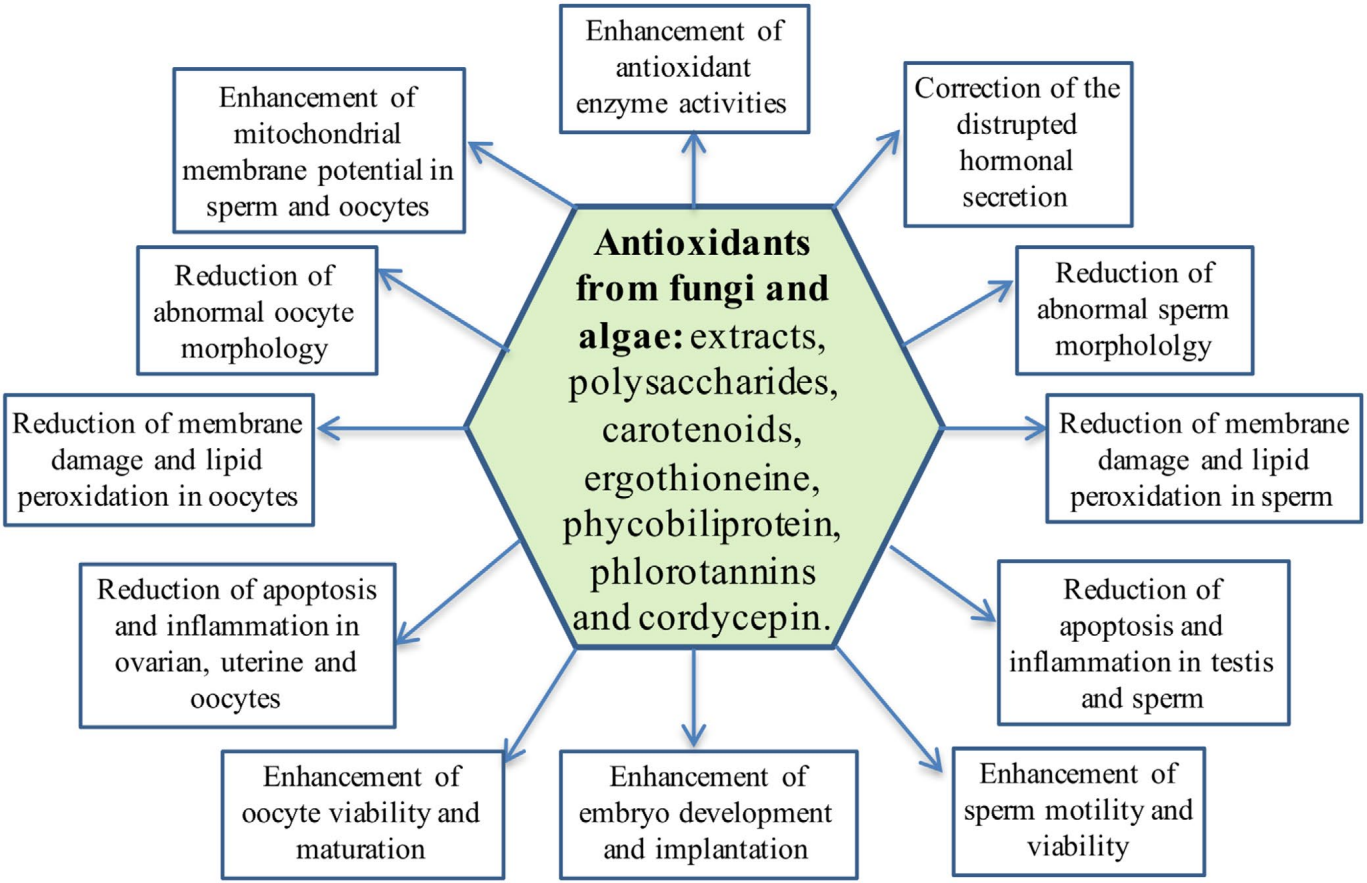
The roles of antioxidants from algae and fungi against oxidative stress‐induced reproductive defects.

The performed studies have revealed that fungi and algae (micro‐and macro‐algae)‐derived metabolites (polysaccharides, carotenoids, ergothioneine, phycobiliproteins, phlorotannins, cordycepin etc.) can prevent or attenuate the oxidative stress‐related reproductive problems in the in vivo and in vitro models (Chen et al. 2010, 2023; Sohn et al. 2012; Kopalli et al. 2019; Pyeon et al. 2021; Ghareeb et al. 2021; Wang, Tan, et al. 2022; Yang et al. 2022; Semaida et al. 2022; Farag et al. 2023; Jeong et al. 2023; Rahim et al. 2024) (Figure 2).

### 5.1 Polysaccharides Against Oxidative Stress‐Induced Reproductive Defects

Polysaccharides are macromolecules consisting of monosaccharide units connected by glycosidic linkages. These macromolecules with high molecular weight are synthesized by all organisms, including plants, animals, fungi, bacteria, macro‐nd microalgae. On the basis of monosaccharide unit, polysaccharides are categorized into two main groups: homopolysaccharides and heteropolysaccharides. The first group includes the polysaccharides consisting of the repeating units of only one type of monosaccharide, whereas those consisting of the repeating units of two or more types of monosaccharides are present in the second group (Arslan et al. 2023; Arslan, Orak, et al. 2024).

Based on their distribution and localization, fungal‐derived polysaccharides are categorized into two main groups: intracellular (IPS) and extracellular polysaccharides (EPSs), and microalgae‐derived ones are categorized into three main groups: structural (cell wall) polysaccharides, reserve polysaccharides (α‐ and β‐glucans) and EPSs (Laroche 2022; Wang, Tan, et al. 2022; Synytsya et al. 2023). The polysaccharides derived from fungi and/or microalgae display various bioactive properties, including antioxidative, immunomodulatory, anticancer, hepatoprotective, anti‐inflammatory, anti‐aging, hypolipidemic, anticoagulant, antilipidemic, antiviral, antibacterial, antifungal, and radioprotective activities (Severo et al. 2022; Arslan et al. 2023).

The performed attempts have demonstrated that fungi or microalgae‐derived antioxidant polysaccharides are capable of protecting reproductive system organs (testis and ovarian) and cells (sperm and oocyt) against oxidative stress induced by natural aging and chemicals, thereby reducing fertilization problems (Table 1). For example, Ak Sonat et al. (2018) revealed that when intragastrically applied to male rats under dietary restriction for 14 days, *Euglena gracilis* ‐derived β‐glucan increased the motility and vitality of spermatozoa, reproductive organ weights (testis, vesicula seminalis and epididymis) and seminiferous tubule diameters. In a different work, Wang, Li, et al. (2022) intended to examine the protective effects of mycelium polysaccharides (MMP) of an edible mushroom *Macrolepiota procera* on nonylphenol (NP)‐induced reproductive impairments in male mice. The results revealed that NP treatment led to oxidative stress in testicular tissues, decreased sperm number and testis index, enhanced sperm deformation, abnormal hormone secretion, and pathological injury. On the contrary, MMP administration reduced oxidative stress and reversed the undesired changes in reproductive parameters in NP‐treated mice. Furthermore, MMP administration was determined to improve noticeably autophagy and inflammatory responses and to suppress the Akt/mTOR signaling pathway in testicular tissues. In another work, Zhou et al. (2023) aimed to examine the effects of polysaccharides (PCPs) from the edible mushroom *Poria cocos* on the quality and DNA methylation of the cryopreserved spermatozoa of Shanghai white pigs. In comparison to the control group (PCPs‐free), PCPs treatment improved spermatozoa viability and increased motility, plasma membrane integrity, acrosome integrity, and mitochondrial activity in the frozen–thawed spermatozoa. The results also displayed that when compared to the control, PCPs treatment decreased ROS and MDA levels and increased SOD, catalase, and GSH‐Px activities in spermatozoa. Furthermore, the results revealed that in comparison to the control, PCPs caused decreases in the levels of 5‐methylcytosine, an indicator of spermatozoa DNA methylation. Overall, the authors concluded that the PCPs‐based treatment strategy may be used for the cryopreservation of pig semen. In a previous study, Ding et al. (2020) aimed to evaluate the effects of an edible mushroom *Inonotus obliquus* polysaccharide (IOP) on the defects in reproductive functions of *Toxoplasma gondii*‐infected male mice. The findings of their study revealed that IOP attenuated pathological damage of the testis and enhanced the spermatogenic capacity, serum testosterone (T), luteinizing hormone (LH) and follicular‐stimulating hormone (FSH) levels when administered orally to *T. gondii*‐infected male mice. IOP also reduced the levels of MDA and nitric oxide (NO) but augmented the SOD activity and glutathione (GSH) level. Moreover, IOP was ascertained to suppress apoptosis in testicular cells by reducing the expression of Bcl‐2 associated × protein (Bax) and cleaved caspase‐3. Overall, the study revealed that IOP exhibited anti‐oxidative stress and anti‐apoptotic activity, thereby reversing notably the defects in the reproductive functions of *T. gondii*‐infected male mice.

**TABLE 1:** Polysaccharides against oxidative stress‐induced reproductive defects.

Toxicity factor	Bioactive compound	In vitro/In vivo activity	References
Dietary restriction	β‐glucan from a microalgae *Euglena gracilis*	Dietary restriction reduced the motility and vitality of spermatozoa, the weights of reproductive organs (testis, vesicula seminalis and epididymis) and the diameter of seminiferous tubules, while β‐glucan ameliorated these defects in sperm parameters and reproductive organs (In vivo)	Ak Sonat et al. (2018)
Nonylphenol	Polysaccharide from an edible mushroom *Macrolepiota procera*	Nonylphenol reduced sperm number and testis index, and also caused sperm deformation, abnormal hormone secretion and pathological injury in the in vivo. Addition of polysaccharide attenuated the these toxic effects of nonylphenol (In vivo)	Wang, Li, et al. (2022)
Exposure of semen to cryopreservation	Polysaccharides from an edible mushroom *Poria cocos*	Cryopreservation decreased the viability, the motility, plasma membrane integrity, acrosome integrity, and mitochondrial activity of pig sperm (in vitro), while the polysaccharide attenuated these toxic effects. Polysaccharides also decreased ROS, MDA and DNA methylation levels and increased SOD, catalase and GSH‐Px activities in spermatozoa (In vitro)	Zhou et al. (2023)
*Toxoplasma gondii* infection	Polysaccharides from an edible mushroom *Inonotus obliquus*	The infection reduced the spermatogenic capacity, caused pathological damage in testis, distrupted hormonal secretion, while the polysaccharide exhibited a protective property against these toxic effects. Furthermore, polysaccharide decreased the levels of MDA and NO^•^ and increased the SOD activity and GSH content (In vivo)	Ding et al. (2020)

Abbreviations: CAT, catalase; GPx, glutathione peroxidase; GSH, glutathione; *MDA, malondialdehyde*; NO^•^, nitric oxide radical; ROS, reactive oxygen species; SOD, superoxide dismutase.

### 5.2 Ergothioneine Against Oxidative Stress‐Induced Reproductive Defects

Ergothioneine (EGT) is a water‐soluble thio‐histidine betaine amino acid derived from histidine (Borodina et al. 2020). Although EGT is synthesized by fungi (mushrooms, yeasts, and molds) and bacteria, its primary producers are mushrooms (Arslan et al. 2023). For example, several mushrooms (*Lentinus edodes, Pleurotus ostreatus, P* *. eryngii* , *Grifola frondosa* etc.) as well as some bacteria ( *Mycobacterium smegmatis* and *Lactobacillus reuteri* ) and the yeasts ( *Rhodotorula mucilaginosa* DL‐X01 and *Aureobasidium pullulans*) can produce naturally EGT (Dubost et al. 2006; Sao Emani et al. 2013; Fujitani et al. 2018; Matsuda et al. 2020; Dare et al. 2021; Pang et al. 2022; Xiong et al. 2023). Moreover, EGT is reported to be produced by genetically engineered some bacteria (*Esherichia coli* and *Methylobacterium aquaticum* strain 22A, *Mycolicibacterium neoaurum*, *Corynebacterium glutamicum* and *Burkholderia pseudomallei* ), yeasts ( *Saccharomyces cerevisiae* , *Schizosaccharomyces pombe*, and *Yarrowia lipolytica*), and filamentous fungus *Aspergillus oryzae* (Gamage et al. 2018; Borodina et al. 2020; van der Hoek et al. 2022; Xiong et al. 2022; Hirasawa et al. 2023). So far, no higher eukaryotes, including humans and plants, have been documented to biosynthesize EGT (Borodina et al. 2020; van der Hoek et al. 2022).

Animals take up EGT with diet from foods (such as mushrooms, grains, internal organs) and then transport it into cells and tissues via specific ergothioneine transporter (carnitine/organic cation transporter OCTN1) on cell membrane (Fu and Shen 2022). It has been documented in the literature that when EGT is administered to humans or animals, it accumulates in some tissues and organs. Even, it is present in human extracellular fluids and secretions, and animal seminal fluids (Halliwell et al. 2018). In the human body, EGT levels are the highest in the liver and erythrocytes; however, its massive accumulation is also high in the intestines, semen, testis, bone marrow, kidney, spleen, lung, eye, and the brain. Especially in the tissues and organs where oxidative stress and inflammation are excess, EGT levels are higher (Fu and Shen 2022).

EGT is considered one of the most powerful antioxidants. It has the potential to scavenge OH^−^, ^1^O_2_, hypochlorous acid (HOCl), and peroxynitrite (ONOO^−^) (Cheah and Halliwell 2012; Fu and Shen 2022). For example, EGT is reported to have higher free radical‐scavenging potency when compared to other well‐known antioxidants, including GSH, Trolox (analogue of vitamin E) and uric acid (Franzoni et al. 2006). Moreover, EGT exhibits other biological activities, namely anti‐inflammatory, anti‐apoptotic, and anti‐aging activities (Apparoo et al. 2022). Because of its antioxidative capacity, it exhibits a strong cytoprotective property in some important cells and tissues. For example, EGT protects erythrocytes against HClO damage (Cheah and Halliwell 2012). Similarly, it protects neurons against toxicity induced by chemicals or drugs (Paul 2022). Additionally, because of the antioxidant and anti‐inflammatory potentials, it exerts a protection towards oxidative stress‐induced diseases, such as diabetes, cardiovascular diseases, liver diseases, neurodegenerative diseases, Crohn's disease, rheumatoid arthritis, and the inflammatory bowel diseases (Fu and Shen 2022). Moreover, the studies have elucidated that the molecule contributes to the reduction of reproductive defects caused by oxidative stress or inflammation (Table 2). For instance, Dare et al. (2019) showed that when cisplatin was applied to male wistar rats, it decreased sperm count, serum testosterone concentration, and the levels of antioxidant enzymes (SOD, GPx, CAT), enhanced abnormal sperm morphology, dead sperm cell number, MDA level, and also led to histopathological damage in the testis, whereas orally applied EGT significantly prevented these deleterious effects of cisplatin. In a different work, Chen et al. (2023) focused on evaluating the therapeutic potential of EGT on the testicular damage induced by varicocele (VC) in vivo, in vitro, and in silico. The results from the in vivo experiments revealed that EGT boosted the sperm quality in VC model rats when administered intragastrically. The in vitro experiments displayed that H_2_O_2_‐induced oxidative stress reduced proliferation and enhanced apoptosis in GC1 and GC2 cells, but the addition of EGT corrected these abnormalities. The molecular docking, western blot, immunohistochemistry, and RT‐qPCR analysis revealed that EGT attenuated the testicular injury by upregulating the expression of the pivotal gene, HSP90AA1. In a recent study, Jeong et al. (2023) examined the influences of EGT addition on both porcine oocyte maturation in the in vitro maturation (IVM) period and embryonic development competence after in vitro fertilization (IVF). The analysis revealed that the EGT group possessed a higher intracellular GSH level and a lower intracellular ROS level after IVM in comparison to the control. Furthermore, EGT was determined to increase the expression levels of the antioxidative pathway‐related genes in cumulus cells and oocytes after IVM. After IVF, the cleavage and blastocyst development formation rates were found to be notably higher in the 10 μM EGT group in comparison to the control group. In a different study (Usuga et al. 2021), the researchers intended to assess the cryoprotective capacity of EGT on the quality of thawed canine semen. The experiments indicated that when compared with controls, EGT (100 μM) increased total and progressive motility, straight line velocity, rapid sperm, and acrosomal integrity and decreased the abnormal morphology and ROS production. Overall, the researchers concluded that ergothioneine attenuates sperm defects and oxidative stress in thawed canine semen.

**TABLE 2:** Ergothioneine against oxidative stress‐induced reproductive defects.

Toxicity factor	Bioactive compound	In vitro/In vivo activity	References
Cisplatin (Chemotherapeutic agent)	Ergothioneine	Cisplatin decreased sperm count, serum testosterone concentration and the activities of antioxidant enzymes (SOD, GPx, CAT) and enhanced abnormal sperm morphology, MDA level and also led to histopathological damage in testis of rats, while orally applied ergothioneine prevented these deleterious effect	Dare et al. (2019)
Varicocele	Ergothioneine	Varicocele reduced the sperm quality and caused testicular injury in rats, while ergothioneine attenuated these deleterious effect (in vivo)	Chen et al. (2023)
Exposure of semen to cryopreservation	Ergothioneine	Cryopreservation process reduced motility, straight line velocity, and acrosomal integrity and increased the abnormal morphology and ROS production in canine semen, but ergothioneine attenuates these deleterious effects	Usuga et al. (2021)

Abbreviations: CAT, catalase; GPx, glutathione peroxidase; *MDA, malondialdehyde*; ROS, reactive oxygen species; SOD, superoxide dismutase.

### 5.3 Cordycepin Against Oxidative Stress‐Induced Reproductive Defects

*Cordyceps militaris* is a medicinally important mushroom species which is widely employed in East Asian countries to treat different diseases and health problems. The mushroom is also used to heal the aging and senescence‐associated health problems, such as weakness in the loins and knees, impotence and seminal emission, hyposexuality, fatigue, and night sweating (Ji et al. 2009; Ramesh et al. 2012). Cordycepin (3′‐deoxyadenosine), an adenosine analog, is a water‐insoluble organic compound synthesized by this mushroom species. It displays various bioactive properties, such as antioxidant, anti‐inflammatory, anti‐aging, anti‐bacterial, antifungal, anti‐malarial, anti‐hyperuricemic, antiviral, antidiabetic, anticancer, immunostimulating, hypolipidemic, anti‐osteoporosis, anti‐arthritic, and hypoglycemic activities (Ng and Wang 2005; Ramesh et al. 2012; Jędrejko et al. 2021). Due to the antioxidant activity, it decreases age‐related oxidative stress and neurodegenerative disorders (Ramesh et al. 2012; Soraksa et al. 2023). Furthermore, some recent studies have proven that cordycepin protects reproduction system organs and/or cells from oxidative stress and inflammation induced by aging or chemicals, thereby attenuating the defects in reproductive functions in animals and humans (Sohn et al. 2012; Kopalli et al. 2019, 2022; Huang et al. 2022; Li, Zhu, et al. 2023; Li, Han, et al. 2023; Li, Wang, et al. 2023) (Table 3). For example, Sohn et al. (2012) focused on testing the useful influences of orally applied cordycepin on the reduced testicular functions caused by aging. The experiments revealed that there were notable decreases in epididymal weight, sperm motility, and spermatogenesis in middle‐aged rats (control group) as compared to the young group. On the contrary, the cordycepin application was determined to prevent the loss in epididymal weights of middle‐aged rats and to improve calcium levels and decrease the levels of urea, nitrogen, uric acid, and creatinine in the blood of middle‐aged rats. Furthermore, this compound was found to boost sperm motility and the progressiveness of sperm movement. Histopathological observations revealed that cordycepin application provided a well‐arranged spermatogonia and densely packed cellular material and enhanced the number of mature spermatozoa in the seminiferous lumen. In a similar work, Kopalli et al. (2019) focused on investigating the protective effects of cordycepin against aging‐induced reproductive defects in male rats. The experiments revealed that the aged‐control group (12‐month‐old; AC) had a diminished sperm quality and altered testicular histomorphology in comparison to young control (2‐month‐old; YC). On the contrary, in comparison to the AC group, the cordycepin (COR)‐treated aged group displayed significant ameliorations in sperm parameters. The COR‐20 mg/kg group was determined to restore the levels of spermatogenesis‐related proteins, histone deacetylating SIRT1 and autophagy‐related mTORC1 in comparison with the AC group. Moreover, the expression levels of key antioxidative enzymes were higher in the COR‐20 mg/kg group in comparison to the AC group. Overall, the researchers informed that cordycepin could ameliorate aging‐mediated testicular dysfunction in rats. In another study, the same research team (Kopalli et al. 2022) tested the potential beneficial influences of cordycepin (COR) on aging‐related testicular inflammation and serum biochemical changes in naturally aged rats. The experiments revealed that there were notable alterations in total blood cell count, lipid metabolism, and liver functional parameters in the aged group in comparison to the young group. Furthermore, there were notable increases in the expression of inflammatory mediators in the testis of aged rats in comparison to young rats. However, cordycepin treatment was determined to ameliorate the alterations in the biochemical parameters and attenuate the increased expression of inflammatory mediators. Another research group (Huang et al. 2022) intended to appraise the possible protective effects of cordycepin administration towards type 2 diabetes‐induced testicular injury in male mice. The results revealed that cordycepin attenuated the testicular damage, repaired the disruption in the blood‐testis barrier, and boosted spermatogenic function via the antiapoptotic and antioxidant capacity. In terms of mechanism, this compound was determined to boost SIRT1 expression, trigger the activity of Foxo3a, and enhance the expression of antioxidant enzymes. In a newly published research, Li, Zhu, et al. (a); Li, Han, et al. (2023); Li, Wang, et al. (2023) demonstrated that cordycepin decreased oocyte fragmentation, corrected spindle/chromosomes morphology and mitochondrial function in oocytes during in vitro postovulatory aging, and increased embryonic developmental potential of oocytes.

**TABLE 3:** Cordycepin against oxidative stress‐induced reproductive defects.

Toxicity factor	Bioactive compound	In vitro/In vivo activity	References
Aging	Cordycepin	Aging process reduced epididymal weight, sperm motility, and spermatogenesis in male rats, whereas orally applied cordycepin attenuated these defects (In vivo)	Sohn et al. (2012)
Aging	Cordycepin	Aging process reduced the motility and progressiveness of sperm as well as the expression levels of SIRT1, antioxidant enzymes (GPx4, GSTm5, and PRx4) and spermatogenesis‐related proteins (CREB‐1, nectin‐2, and inhibin‐α) in male rats. It also increased the level of autophagy‐related protein (mTORC1). On the contrary, cordycepin could ameliorate or reverse these abnormalities (In vivo)	Kopalli et al. (2019)
Aging	cordycepin	Aging process increased the levels of inflammatory mediators (COX‐2, IL‐6, IL‐1β, and TNF‐α) in testis of rats and caused the significant alterations in the total blood cell count, lipid metabolism, and liver functional parameters in rats, while cordycepin treatment prevented or attenuated these abnormalities (In vivo).	Kopalli et al. (2022)
Diabetes‐induced toxicity	Cordycepin	Diabetes‐induced toxicity increased apoptosis and reduced antioxidant capacity (Mn‐SOD and CAT) and SIRT1 expression in male mice model, thereby causing the testicular damage and reducing spermatogenic function, whereas cordycepin prevented or reversed these abnormalities (In vivo)	Huang et al. (2022)
In vitro postovulatory aging	*B. amyloliquefaciens* SJ100001	The postovulatory aging increased oocyte fragmentation and abnormal spindle/chromosomes morphology and reduced mitochondrial function, while cordycepin ameliorated these abnormalities (In vitro)	Li, Zhu, et al. (2023); Li, Han, et al. (2023); Li, Wang, et al. (2023)

Abbreviations: CAT, catalase; COX‐2, cyclooxygenase 2; CREB1, cAMP responsive element binding protein 1; GPx, glutathione peroxidase; GPx4g, glutathione peroxidase; GSTm5, glutathione S‐transferase mu 5; IL‐1β, interleukin‐1 beta; IL‐6, interleukin 6; Mn‐SOD, manganese superoxide dismutase; mTORC1, mammalian target of rapamycin complex 1; PRx4, peroxiredoxin; SIRT1, sirtuin 1; TNF‐α, tissue necrosis factor‐alpha.

### 5.4 Phycobiliproteins Towards Oxidative Stress‐Induced Reproductive Defects

Phycobiliproteins (PBPs) are water‐soluble proteins that are present in various groups of algae (cyanobacteria, eukaryotic microalgae and eukaryotic macroalgae) and account for about 40%–60% of all soluble proteins in their cells. In these organisms, PBPs function as the main building blocks of light‐harvesting complexes. According to light absorption characteristics, these proteins are classified into four groups: phycoerythrins (pink‐purple), phycoerythrocyanins (orange), phycocyanins (blue), and allophycocyanins (Pan‐utai and Iamtham 2019; Chen et al. 2022; Dagnino‐Leone et al. 2022; Esim et al. 2024). Because of some bioactive properties, they have also a high potency to be utilized in the cosmetic, pharmaceutical, and nutraceutical industries (Li et al. 2016; Yang et al. 2022; Dagnino‐Leone et al. 2022). For example, the in vitro and in vivo works have clarified that PBPs can be effective to reduce oxidative stress‐induced reproductive defects (Table 4). A previous research (Montaño‐González et al. 2022) appraised the protective capacity of orally applied *Spirulina* (Sp) and its PBP extract against toxic effects of Cd on the testicular tissues and spermatozoa in male mice. The experiments demonstrated that PBP and Sp alleviated the Cd toxicity on sperm motility and viability. Furthermore, PBP was ascertained to reduce Cd toxicity‐induced MDA production in the spermatozoa. Within the seminiferous tubule, Cd caused necrosis and apoptosis‐related areas, but PBPs and Sp improved them. Overall, the research team informed that PBPs have the capacity to protect testes and sperm againstCd‐induced toxicity. A different in vivo study (Boukari et al. 2024) was conducted to examine the ability of *Spirulina platensis*‐derived C‐phycocyanin to ameliorate the toxicity of ethanol on reproductive parameters in male rats. The findings revealed that ethanol exposure decreased sperm production and viability, altered testicular weight and morphology, and enhanced lipid peroxidation when applied to male rats, whereas orally given C‐phycocyanin hindered these detrimental effects and sustained normal sperm production and viability. In a newly published study, Rahim et al. (2024) revealed that C‐phycocyanin from *S. platensis* improved ram semen quality parameters (total motility, viability etc.) in Boujaâd rams during cooling storage at 5°C. The data also displayed that C‐phycocyanin enhanced the in vivo fertilization potential of ram semen. Yang et al. (2022) revealed that TNF α + Smac mimetic + zVAD‐fmk (TSZ) treatment reduced cell viability and increased cell death in the in vitro model of GC‐1 spg cells, whereas C‐phycocyanin from *Spirulina platensis* could attenuate these defects. Similarly, the in vivo experiments of the study elucidated that orally applied C‐phycocyanin protected the reproductive system of male mice against cyclophosphamide‐induced toxicity, improved spermatogenic activity, sperm quality, and fertilization success, enhanced testosterone production, and stabilized the feedback regulation mechanism. A previous in vivo work (Li et al. 2016) was undertaken to examine the potency of C‐phycocyanin from *Spirulina platensis* to reverse the negative effects of D‐galactose‐induced aging process on the reproductive ability of adult female B6D2F/1 mice. The results displayed that C‐phycocyanin could restrict oocyte fragmentation and aneuploidy induced by aging. Furthermore, C‐phycocyanin was ascertained to inhibit ROS production, enhance antioxidant activity, and normalize mitochondria distribution. Overall, the researchers exhibited that C‐phycocyanin can reduce the accumulation of ROS and prevent partially the negative effects of D‐galactose‐induced aging process on female reproductive ability. Another research group (Wen et al. 2020) intended to assess the ability of C‐phycocyanin from *Spirulina platensis* in reversing the detrimental effects of obesity on fertilization. They found that C‐phycocyanin administration to obese mice was able to increase litter size and offspring survival rates, ameliorate the level of ovarian antioxidant enzymes, decrease follicular atresia, and correct the abnormal morphology of the spindle‐chromosome complex and the abnormal mitochondrial distribution pattern in oocytes. Moreover, C‐phycocyanin treatment was found to decrease the obesity‐related ROS accumulation and early apoptosis in oocytes. In a newly published study, Wang, Liu, et al. (2024) reported that C‐phycocyanin maintains regular the morphological features of cryopreserved human oocytes and improves their maturation by decreasing intracellular ROS production and early apoptosis rate and enhancing the mitochondrial membrane potential. Furthermore, they reported that C‐phycocyanin enhanced the cleavage and blastocyst formation of vitrified oocytes after in vitro maturation and parthenogenetic activation. A different research team (Wang, Gao, et al. 2024) focused on investigating the influences of C‐phycocyanin on the in vitro maturation of goat oocytes or developmental competence after vitrification. They found that C‐phycocyanin addition into the oocyte maturation medium facilitated the first polar body extrusion, cumulus expansion index, parthenogenetic blastocyst formation, cortical granules migration, mitochondria distribution, and mitochondrial membrane potential but inhibited the reactive oxygen species accumulation and cell apoptosis.

**TABLE 4:** Phycobiliproteins and phlorotannins against oxidative stress‐induced reproductive defects.

Toxicity factor	Bioactive compound	In vitro/In vivo activity	References
Heavy metal (Cd)	Phycobiliproteins	Cd‐induced toxicity caused necrosis and apoptosis in seminiferous tubule and also reduced the sperm motility and viability and increased MDA content, while phycobiliproteins‐extract attenuated these deleterious effects (in vivo)	Montaño‐González et al. (2022)
Cooling storage	C‐phycocyanin	Cooling storage reduced ram semen quality parameters (motility, progressive motility, curvilinear velocity, straight‐line velocity, average path velocity and viability) in Boujaâd rams, while C‐phycocyanin attenuated these deleterious effects induced by cooling storage (in vitro)	Rahim et al. (2024)
Cyclophosphamide	C‐phycocyanin	Cyclophosphamide reduced spermatogenesis, sperm quality, fertility, and testosterone level in mice, but orally applied C‐phycocyanin attenuated these negative effects (In vivo)	Yang et al. (2022)
Ethanol	C‐phycocyanin	Ethanol decreased sperm production and viability, caused testicular damage, and enhanced lipid peroxidation in male rats, but the oral supplementation of C‐phycocyanin ameliorated these damages (In vivo)	Boukari et al. (2024)
Aging	C‐phycocyanin	D‐galactose‐induced aging process caused oocyte fragmentation, aneuploidy, ROS accumulation, abnormal mitochondrial distribution, and the reduced antioxidant mechanism in female mice, while C‐phycocyanin reversed these defects (In vivo)	Li et al. (2016)
Obesity	C‐phycocyanin	Obesity reduced offspring survival rates and ovarian antioxidant enzyme activites, and increased follicular atresia, ROS accumulation, early apoptosis, and abnormal mitochondrial distribution in oocytes. While, C‐phycocyanin attenuated or reversed these abnormalities (In vivo)	Wen et al. (2020)
Cryopreservation	C‐phycocyanin	Cryopreservation process increased intracellular ROS and early apoptosis rate, and reduced the mitochondrial membrane potential in human oocytes, while C‐phycocyanin reversed these defects and also blastocyst formation of vitrified oocytes after in vitro maturation	Wang, Liu, et al. (2024)
	Dieckol (a phlorotannin)	Oxidative stress increased apoptotic cells and decreased the blastocyst formation in the *in* IVM medium, while Dieckol could increase the level of glutathione and expression of antioxidant genes (*NFE2L, SOD1*, and *SOD2*) and reduce ROS, thereby preventing these deletirous effects of oxidative stress (In vitro)	Pyeon et al. (2021)

Abbreviations: IVM, in vitro maturation media; *MDA, malondialdehyde*; *NFE2L*, Nuclear factor erythroid 2‐related factor 2; ROS, reactive oxygen species; SOD1 and SOD2, superoxide dismutase 1 and 2.

### 5.5 Phlorotannins Against Oxidative Stress‐Induced Reproductive Defects

Phlorotannins are phenolic compounds with antioxidant activity, which are extracted from marine macroalgae, mainly from brown macroalgae. Based on the degree of polymerization and structural diversities, these can be classified into six different groups: phloroethols, fuhalols, fucophloroethols, fucols, eckols, and carmalols. Their function is to protect algae against challenging aquatic conditions (Priyanka et al. 2022; Kumar et al. 2022; Esim et al. 2024). Moreover, phlorotannins derived from algae are useful for humans due to their health‐promoting properties, such as antioxidative, antibacterial, antiviral, anticancer, anti‐hypertensive, hypoglycemic, anti‐allergic, and anti‐inflammatory activities (Zheng et al. 2022). Especially due to the antioxidant property, phlorotannins have been documented to find applications in oxidative stress‐mediated health problems, such as neurodegenerative diseases, hepatic diseases, diabetes, and retinal diseases (Phang et al. 2023). Moreover, in a study (Pyeon et al. 2021), the researchers elucidated the beneficial effect of the dieckol, a phlorotannin of an edible brown macroalgae *Ecklonia cava*, on maturation and developmental competence of porcine oocytes exposed to oxidative stress in vitro. The experiments demonstrated that 0.5 μM dieckol reduced the ratio of apoptotic cells and augmented the blastocyst formation in the in vitro maturation (IVM) medium supplemented with 0.5 μM dieckol. Moreover, 0.5 μM dieckol was found to decrease ROS, increase the antioxidant capacity, and prevent abnormal spindle organization and chromosome misalignment as well as enhance expression of maternal markers (*CCNB1* and *MOS*) and activity of p44/42 mitogen‐activated protein kinase (Table 4).

### 5.6 Carotenoids Against Oxidative Stress‐Induced Reproductive Defects

Carotenoids are lipophilic pigments that are produced by plants, algae, archaea, yeasts, fungi, and some animals (insects, mites, fish, birds, etc.). The two main types of carotenoids are carotenes and xanthophylls. The most‐known ones of carotenes are α‐, β‐, and γ‐carotene, torulene, and lycopene. The examples of xanthophylls include zeaxanthin, astaxanthin, lutein, *β*‐cryptoxanthin, fucoxanthin, and peridinin. Carotenoids, namely carotenes and xanthophylls, are employed as natural colorants in the food industry. Furthermore, because of different biological activities, they find numerous practices in the pharmaceutical, nutraceutical, and cosmetic industries (Dewanjee et al. 2021; Arslan et al. 2023; Gebregziabher et al. 2023; Esim et al. 2024). For example, they exhibit a protective role against diverse health disorders, such as infertility, neurodegenerative diseases, age‐related macular degeneration and cataract, non‐alcoholic fatty liver diseases, and cardiovascular disease (Kong et al. 2019; Jia et al. 2020; Kabir et al. 2022; Gebregziabher et al. 2023).

Eventhough astaxanthin can be synthesized by diverse organisms, such as molds, microalgae, animals, it's commercial production is performed using the yeasts *Phaffia rhodozyma and Xanthophyllomyces dendrorhous*, and *the microalgae Haematococcus pluvialis* (Patel et al. 2022; Ritu et al. 2023; Arslan et al. 2023). Fucoxanthin is another xanthophyll‐type carotenoid found in marine algae, such as brown macroalgae and microalgae. This pigment acts as a light‐harvesting complex for photosynthesis and photoprotection (Peng et al. 2011; Din et al. 2022). In addition to their roles in the organisms in which they are found, astaxanthin and fucoxanthin also exhibit numerous health beneficial properties for humans (Peng et al. 2011; Fakhri et al. 2018). Due to the antioxidative and anti‐inflammatory activities, astaxanthin and fucoxanthin can protect reproductive system organs and cells towards oxidative damage (Table 5). For instance, Jang et al. (2010) exhibited that the astaxanthin in a dose‐dependent manner alleviated the nitric oxide‐induced oxidative stress (lipid peroxidation), upregulated antioxidant genes and downregulated apoptotic genes, thereby decreasing the negative effect of the oxidative stress on the cell viability of bovine oviduct epithelial cell (BOEC) and the development potential of bovine IVM/IVF embryos. A different study (Kuroki et al. 2013) elucidated that the supplementatoln of astaxanthin into the medium can reduce the developmental defects of bovine embryos cultured in the in vitro under heat stress, recover the mitochondrial membrane potential of embryos and provide a notable recovery in blastocyst development. Jia et al. (2020) revealed that the astaxanthin alleviated oxidative stress in aged oocytes in the in vitro by decreasing ROS and enhancing glutathione and antioxidant gene expression. They also reported that the astaxanthin inhibited apoptosis and autophagy in aged oocyte, maintained spindle organization and actin expression, rescued functional status of organelles, and restored the quality and developmental competency of aged porcine oocytes. Overall, they suggested that the astaxanthin can be useful for assisted reproductive technologies due to its protective potency against oocyte aging. In a work performed by Gao et al. (2021), the influences of dietary astaxanthin (from *Haematococcus pluvialis* ) on semen quality (sperm viability, motility, and concentration) and antioxidant potential in aged‐roosters were tested. The experiments revealed that the dietary astaxanthin increased antioxidant enzyme levels and showed more radical‐scavenging capacity via the upregulation of the MAPK/Nrf2 pathway, thus improved the semen quality. Another research group (Guo et al. 2021) made an effort to reveal whether the astaxanthin from *H. pluvialis* preserves boar sperm against ROS‐induced oxidative stress during cryopreservation process. They found that the supplementation of astaxanthin to freezing extenders, specially at a concentration of 2 μM, enhanced sperm motility, membrane integrity, and acrosome integrity, prevented lipid peroxidation, and corrected the fatty acid composition of the sperm membrane. Kong et al. (2019) reported that when the fucoxanthin from brown algae *Laminaria japonica* was orally given into the streptozotocin‐induceddiabetic rat model, it improved insulin resistance, exhibited antioxidant and anti‐inflammatory properties, increased sperm motility, reduced abnormal sperm number, prevented lipid peroxidation, and restored luteinizing hormone and testosterone levels, and eventually improved reproductive functions. Wang et al. (2020) demonstrated that the administration of chemotherapeutic drug cisplatin into hamsters led to sperm abnormalities and distrupted the seminiferous tubules morphology and testosterone levels, whereas the oral administration of fucoxanthin extract from an edible brown algae *Sargassum glaucescens* prevented or reversed these defects.

**TABLE 5:** Carotenoids against oxidative stress‐induced reproductive defects.

Toxicity factor	Bioactive compound	In vitro/In vivo activity	References
Nitric oxide	Astaxanthin	Nitric oxide caused lipid peroxidation, decreased the expression of antioxidant genes (*CuZnSOD, MnSOD* and *Catalase*) and increased the expression of apoptotic genes (*Caspase‐3* and *Bax* genes) in bovine oviduct epithelial cell, but astaxanthin treatment reversed or attenuated these abnormalities (In vitro)	Jang et al. (2010)
Heat stress	Astaxanthin	Heat stress reduced mitochondrial membrane potential and caused developmental defects in bovine embryos cultured in the in vitro, whereas astaxanthin supplementation ameliorated these defects and increased blastocyst development (In vitro)	Kuroki et al. (2013)
In vitro aging	Astaxanthin	The in vitro aging increased ROS, apoptosis and autophagy and caused abnormal spindle organization and Actin expression in oocytes, while astaxanthin treatment reduced ROS and attenuated these abnormalities (In vitro)	Jia et al. (2020)
Aging	Astaxanthin	Aging process reduced increased oxidative sress‐related lipid peroxidation thereby decreasing sperm viability and sperm concentration in roosters, while astaxanthin upregulated MAPK/Nrf2 pathway, increased antioxidant enzyme activities (SOD, CAT, GSH‐Px and T‐AOC) and corrected semen defects (In vivo)	Gao et al. (2021)
Cryopreservation	Astaxanthin	The cryopreservation process increased lipid peroxidation and reduced sperm motility, membrane integrity, and acrosome integrity in boar sperm, while the addition of astaxanthin to freezing extenders improved sperm parameters and inhibited lipid peroxidation (In vitro)	Guo et al. (2021)
Diabetes‐induced toxicity	Fucoxanthin	Diabetes‐induced toxicity reduced sperm motility, increased abnormal sperm number and lipid peroxidation, and caused the alterations in the levels of luteinizing hormone and testosterone, while the fucoxanthin extract exhibited antioxidant and anti‐inflammatory properties and attenuated these abnormalities (In vitro)	Kong et al. (2019)

Abbreviations: CAT, catalase; GSH‐Px, glutathione peroxidase; MAPK, mitogen‐activated protein kinase; Mn‐SOD, manganese superoxide dismutase; Nrf2, nuclear factor erythroid 2‐related factor 2; SIRT1, sirtuin 1; T‐AOC, total antioxidant capacity.

## 6 Limitations of Using Antioxidants in Clinic Studies

To date, many studies have investigated the protective role of natural or synthetic molecules with antioxidant activity against pathologies, such as infertility, the cardiovascular system, aging, and diabetes.

In some of these studies, it has been reported that antioxidant molecules possess therapeutic effectiveness against the pathologies in question, and in some others, on the contrary, they did not provide significant benefits. For example, some preclinical experiments have shown that antioxidants, such as diapocynin, carnosine, and carvacrol may be effective in treating or preventing oxidative stress‐related diseases (Boldyrev et al. 2008; Dranka et al. 2013; Dost et al. 2024). Similarly, some studies have reported that the antioxidants, such as melatonin, CoQ10, NAD^+^ precursors (nicotinamide mononucleotide and nicotinamide riboside) and resveratrol reduce reproductive defects that cause infertility both in vivo (animal or human models) and in vitro (assisted reproduction technology) (Xu et al. 2018; Galano and Reiter 2018; Bahramrezaie et al. 2019; Arslan, Taskin, and Keles 2024). For example, the subcutaneous administration of CoQ10 to mice was reported to protect ovarian reserves against aging, improve mitochondrial functions in the ovaries, and increase the rate of blastocyst development (Boots et al. 2016; Delkhosh et al. 2019). A randomized study performed on humans (Xu et al. 2018) exhibited that the oral administration of CoQ10 to women increased embryo quality and fertilization rate, but caused no prominent advancement in clinical pregnancy and live birth rates per embryo transfer. In another clinical study (Bahramrezaie et al. 2019), resveratrol administered orally to humans was shown to improve oocyte and blastocyst quality but did not affect fertilization success. In a recent study, Arslan, Taskin, and Keles (2024) reported that nicotinamide riboside and nicotinamide mononucleotide rebalanced mitochondrial dynamics and activated SIRT1, thus reversing ovarian aging in middle‐aged rats, alleviating mitochondrial stress, and correcting aging‐induced folliculogenesis abnormalities. Some in vitro studies demonstrated that the addition of CoQ10 or NAD+ precursors to the culture media increased reduced oxidative stress, increased oocyte maturation, and blastocyst development (Al‐Zubaidi et al. 2021; Wang et al. 2023; Khan et al. 2023; Li, Zhu, et al. 2023; Li, Han, et al. 2023).

On the contrary, some studies have reported that antioxidant molecules (glutathione, N‐acetylcysteine, β‐carotene, vitamin E, vitamin C, lycopene, lutein, quercetin, routine, catechins, selenium, retinol, zinc, riboflavin, CoQ10, molybdenum etc.) do not have a significant protective role against oxidative stress‐induced pathologies, such as infertility, cancer, diabetes, and neurodegenerative diseases (Iannitti and Palmieri 2009; Goodman et al. 2011; Bjelakovic et al. 2012; Bentov et al. 2014; Murphy 2014; Caballero et al. 2016; Forman and Zhang 2021; Banerjee et al. 2022; Pappolla et al. 2024). For example, it has been stated that CoQ10 does not statistically affect oocyte numbers, implantation rate, or clinical pregnancy rate in humans (Bentov et al. 2014; Caballero et al. 2016). Evenso, a previous study reported that orally applied resveratrol reduced clinical pregnancy rate and enhanced the risk of miscarriage (Ochiai et al. 2019).

A major limitation of using the antioxidants in the treatment of pathologies is the difficulty in delivering enough of the antioxidants to the targeted intracellular location, for example, preferentially inside mitochondria (Murphy 2014; Banerjee et al. 2022). To solve this problem, conjugates that transport antioxidants to target organs have been prepared. For example, for the treatment of Parkinson's disease, antioxidants have been combined with triphenylphosphonium (TPP+), which has the capacity to pass the blood–brain barrier and target mitochondria, and the prepared conjugates have been tested in animal models either orally or intraperitoneally. Obtained preclinical results revealed that mitochondria‐targeted antioxidants conjugated with TPP+ provide better protection against Parkinson's disease compared to non‐targeted ones (Yang, Calingasan, et al. 2009; Yang, Zhao, et al. 2009; Ghosh et al. 2010, 2012; Jin et al. 2014; Ramis et al. 2015). Similarly, in preclinical studies, MitoQ10, a mitochondria‐targeted antioxidant, has been shown to have a protective role against autoimmune and cardiovascular diseases (Graham et al. 2009; Mao et al. 2013; McLachlan et al. 2014). Moreover, the effectiveness of targeted antioxidants against infertility factors has been tested. For instance, in a performed study (Ding et al. 2019), the therapeutic effectiveness of the mitochondria‐targeted antioxidant MitoQ10 against polycystic ovary syndrome (PCOS) was tested in female Sprague–Dawley rats. The results of the study revealed that MitoQ10 significantly improved insulin resistance, reduced apoptosis, and also restored the endocrine functions, reproductive conditions, and mitochondrial activity.

In short, convincing findings about the effectiveness of plant‐derived antioxidants or synthetic antioxidant molecules have not been obtained. Furthermore, overdoses of some antioxidants, such as β‐carotene, and vitamins C and E have been reported to cause reductive stress, thereby contributing to the development of some pathologies (Pérez‐Torres et al. 2017; Al‐Madhagi and Masoud 2024). For these reasons, the clinical use of antioxidants is still controversial. Even so, new studies are being carried out to reveal the therapeutic potential of currently known antioxidant molecules or newly discovered ones. For instance, there are numerous studies on the protective effects of fungal or algal antioxidants against the in vivo and in vitro reproductive defects caused by natural aging and environmental factors (Table 1). However, it should be noted that their effectiveness was tested in only animal models and not on humans. Therefore, the same success may not be achieved in human models. Besides, the toxicity analysis of algal and fungal antioxidants has not been performed in detail in any of the animal studies summarized in Table 1. Accordingly, further animal and human studies should be conducted to elucidate the potential toxicities of fungal or algal antioxidant compounds. To increase the effectiveness of fungal or algal antioxidants and to minimize their side effects, these antioxidants can be modified to target specific organs or organelles. For example, these antioxidants can be conjugated with TPP^+^ to target the mitochondria. Furthermore, although there are commercial formulations of natural products obtained from fungi and algae sold as supplements (Niego et al. 2021; Matos et al. 2022), their effectiveness has not been tested against reproductive defects or on the fertilization success in the in vivo or in vitro models. Therefore, the commercial formulation of fungi or algae‐derived compounds should also be tested in terms of their useful or detrimental influences on human fertilization.

## 7 Conclusion and Future Perspectives

The accumulation of ROS and the occurrence of oxidative damage in reproductive system organs and cells impairs spermatogenesis, ovulation, fertilization, and implantation in humans and animals and eventually causes infertility. The present review reveals that fungi and/or algae, especially edible ones, are important sources of exogenous antioxidants exhibiting protective roles against infertility. In the in vivo models, fungi and/or algae‐derived antioxidants correct the defects in reproductive functions by protecting reproductive system organs and cells from oxidative stress caused by different factors, such as natural aging, diseases, and chemicals. In the in vitro models, they can also protect spermatozoa and oocytes against oxidative stress induced by toxic chemicals or cryopreservation. However, the literature survey revealed that, in contrast to the plant‐derived antioxidants, there is limited research on the protective roles of fungi and algae‐derived antioxidants, especially phlorotannins, against oxidative stress‐induced infertilization. Therefore, we suggest that fungi and algae‐derived antioxidants, especially phlorotannins, should be investigated more in future studies. Overall, this review indicates that the purified forms and extracts of fungal and algal antioxidants have the potential to be used as protective agents against reproductive defects induced by oxidative stress, particularly natural aging. Furthermore, this review exhibits that the antioxidants from fungi and algae can improve the success of assisted reproductive technology. Even so, more in vivo, in vitro, or clinical studies are needed to prove their safety profile and efficacy.

## Author Contributions

**Nazli Pinar Arslan:** conceptualization (lead), investigation (equal), software (equal), supervision (equal), writing – original draft (equal). **Seyda Albayrak:** investigation (supporting), writing – original draft (supporting). **Aysenur Budak‐Savas:** investigation (equal), writing – original draft (equal). **Ahmet Hacimuftuoglu:** investigation (equal), writing – original draft (equal). **Tugba Orak:** investigation (supporting), writing – original draft (supporting). **Omer Karadagoglu:** investigation (supporting), writing – original draft (supporting). **Aysenur Ozdemir:** investigation (supporting), writing – original draft (supporting). **Sevval Yildirim:** investigation (supporting), writing – original draft (supporting). **Handan Cinar‐Yilmaz:** investigation (supporting), writing – original draft (supporting). **Mesut Taskin:** investigation (equal), software (equal), supervision (equal), writing – original draft (lead), writing – review and editing (lead).

## Ethics Statement

The authors have nothing to report.

## Consent

Written informed consent was obtained from all study participants.

## Conflicts of Interest

The authors declare no conflicts of interest.

## Data Availability

No data was used for the research described in the study.
